# High survival rates and patient satisfaction 12 years after medial open wedge high tibial osteotomy surgery: A prospective cohort study

**DOI:** 10.1002/ksa.70071

**Published:** 2025-09-23

**Authors:** Marc‐Daniel Ahrend, Daniel Petzold, Tina Histing, Christoph Ihle, Steffen Schröter, Moritz Herbst

**Affiliations:** ^1^ Department of Traumatology and Reconstructive Surgery, BG Trauma Center Tübingen Eberhard Karls University Tübingen Tübingen Germany; ^2^ Department of Orthopedics m&i‐ Fachkliniken Hohenurach Bad Urach Germany; ^3^ Department of Traumatology and Reconstructive Surgery Diakonie Klinikum GmbH Jung‐Stilling‐Krankenhaus Siegen Germany

**Keywords:** high tibial osteotomy, knee, long‐term follow‐up, osteoarthritis, prospective cohort study

## Abstract

**Purpose:**

Medial open wedge high tibial osteotomy (HTO) can delay knee arthroplasty (KA) in patients with medial compartment varus knee osteoarthritis (OA). However, prospectively collected long‐term outcomes and survival rates are limited. The purpose of this study was to assess the survival rate and the outcome following HTO.

**Methods:**

In this prospective cohort study with initially 120 knees from 112 patients treated from 2008 to 2011 with an HTO, 95 knees from 88 patients (age: 47.0 ± 7.6 years; female: *n* = 28) were followed‐up. The minimum follow‐up was 12 years or an earlier conversion to KA. The 5‐, 10‐ and 12‐year survival rates were calculated. The Lysholm and IKDC scores were assessed preoperatively and 1.5, 6 and 12 years postoperatively.

**Results:**

At the last follow‐up (12.9 ± 0.8,12.0–15.1 years), 67.4% (*n* = 64) had no conversion to KA. 31 knees (32.6%; 2 unicompartmental KA, 29 total KA) were converted to a KA on average 7.3 ± 3.3 (1.5–13.0) years after the HTO. The 5‐, 10‐ and 12‐year survival rates were 88.2%, 76.3% and 69.7%. Knees without conversion to KA had significantly higher scores at the last follow‐up compared to preoperatively: The Lysholm score increased from 60.4 ± 21.1 (14.0–91.0) preoperatively to 89.1 ± 12.5 (39.0–100.0), 86.5 ± 13.8 (39.0–100.0) and 82.6 ± 18.3 (30.0–100.0). The IKDC score also increased from 51.8 ± 16.6 (15.0–93.0) preoperatively to 77.7 ± 14.8 (21.0–100.0), 70.9 ± 15.3 (26.0–98.0) and 72.5 ± 18.1 (14.0–95.0) at the corresponding postoperative time points 1.5, 6 and 12‐years.

**Conclusion:**

HTO to treat varus medial OA showed good long‐term outcomes. Most patients can expect no conversion to KA for more than twelve years and a higher subjective knee function than preoperatively.

**Level of Evidence:**

Level IV.

**Abbreviations**
BMI: body mass index
CI: confidence interval
DRKS: Deutsches Register Klinischer Studien (German Clinical Trials Register)
ESSKA: European Society of Sports Traumatology, Knee Surgery & Arthroscopy
HTO: high tibial osteotomy
IKDC: International Knee Documentation Committee (Score)
JLCA: joint line convergence angle
JLO: joint line orientation
KA: knee arthroplasty
LCP: locking compression plate
mLDFA: mechanical lateral distal femur angle
mMPTA: mechanical medial proximal tibia angle
mTFA: mechanical tibiofemoral angle
n.s.: not significant
OA: osteoarthritis
OR: odds ratio
SD: standard deviation

## INTRODUCTION

Medial open wedge high tibial osteotomy (HTO) is a widespread and commonly accepted surgery to treat patients with medial compartment knee osteoarthritis (OA) with varus malalignment. A primary goal of this surgery is to avoid or delay knee arthroplasty (KA) in the long‐term [7, 11, 27]. Several clinical studies reported good to excellent mid‐ to long‐term results, demonstrating improved quality of life and knee function [15, 21, 22, 32, 36, 42]. However, most findings are based on retrospective study designs with high lost‐to‐follow‐up rates [5, 26, 31, 41]. Furthermore, studies reporting long‐term outcomes include different surgical techniques, such as lateral closed wedge HTO or different plate designs in their analysis [5, 29, 31]. Based on these data, the prediction of the outcome following HTO surgery is difficult. Furthermore, analysis of risk factors of conversion to total knee arthroplasty showed divergent results [14, 18, 33, 43].

Therefore, the purpose of the present study was to analyse the long‐term outcome regarding patient‐reported outcomes and survival rates (endpoint defined as conversion to arthroplasty) following medial open wedge HTO. It was hypothesised that most patients do not receive a KA during a 12‐year follow‐up period and that patients who do not receive a KA still have higher subjective knee function than preoperatively.

The results of the study should help surgeons to better predict long‐term outcomes of HTO and provide further information for realistic patient education as well as improvements of surgical indications.

## MATERIALS AND METHODS

This prospective cohort study (German clinical trials register: DRKS00005614) was approved by the local ethics committee (10/2008BO2, 488/2014BO2, 409/2017BO2, 095/2022/BO2). Informed consent was obtained from all individual participants included in the study. Inclusion criteria were patients with symptomatic varus malalignment and medial compartment osteoarthritis of the knee joints in active patients. All patients with previous or acute infections of the knee joint, osteoporosis, or those aged under 18 years were excluded. Between 2008 and 2011, 120 knees from 112 patients underwent a medial open wedge HTO and were included in this prospective clinical trial. Eight patients received HTO surgery on both knees and were included into the study with both surgeries. Time betweeen surgeries was in average 15.1 months (minimum: 7.9–maximum: 30.7). The surgery was based on a landmark‐based deformity analysis. A high biplanar opening wedge HTO for valgisation with a locking plate (TomoFix™), as described by Staubli et al. as well as Lobenhoffer and Agneskirchner was performed in all knees [12, 25, 40]. The correction of the varus alignment was aimed to 1°–3° of valgus. The osteotomy gap was not filled with bone graft or substitute [25, 40]. Half of the cases received partial postoperative weight bearing for 2 weeks, and the other half of the cases for 6 weeks based on the initial study's protocol. Postoperative management and further details concerning the performed procedure and study protocols are described in previous publications [16, 17, 19, 37, 38].

**Figure 1:**
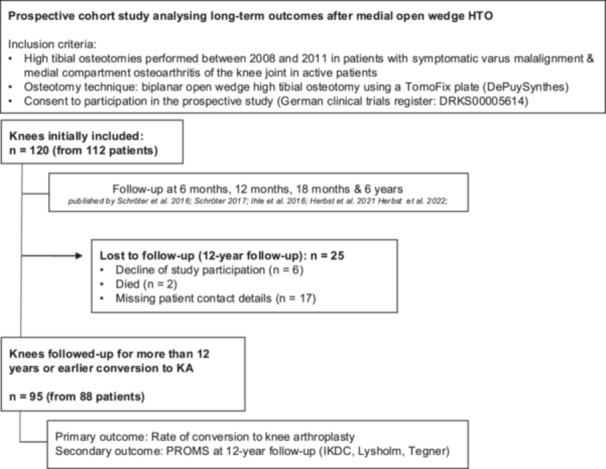
Flow chart. HTO, high tibial osteotomy; IKDC, International Knee Documentation Committee (Score); KA, knee arthroplasty.

All 120 knees were included in this prospective clinical trial. Knees were examined preoperatively and 6 weeks postoperatively, directly after reaching full weight bearing. The further follow‐ups took place at 6 months, 12 months, 18 months, and 6 years after surgery. These results were previously published in [16, 17, 19, 37, 38]. A last follow‐up of this study was conducted between 2021 and 2024, at least 12 years after surgery, for the purpose to assess the long‐term outcome following HTO. 25 cases were lost‐to‐follow‐up due to declined study participation (*n* = 6), death (*n* = 2) and missing patient contact details (*n* = 17) (Figure 1). From the included patients, seven received HTO surgery on both knees. Resultingly, the final cohort consisted of 95 knees from 88 patients.

Preoperatively and at each follow‐up, the clinical outcome was evaluated using established and standardised instruments (Lysholm, IKDC, Tegner Activity Score). Furthermore, full weight‐bearing long‐leg anteroposterior radiographs were obtained preoperatively and 6 weeks postoperatively to assess the postoperative alignment. The mechanical tibiofemoral angle (mTFA), the mechanical lateral distal femur angle (mLDFA), the joint line convergence angle (JLCA), and the medial proximal tibia angle (mMPTA) were measured with an accuracy of one decimal place using digital planning software (mediCAD, Hectec, Landshut, Germany).

The primary outcome parameter was the rate of knees with conversion to KA and the time between index surgery and conversion. Patient characteristics (gender, age, height and weight), preoperative deformity analysis, and postoperative alignment parameters were collected. The IKDC score and the Lysholm score were used to assess the change in subjective knee function at each follow‐up time point.

### Statistical analysis

A Kaplan–Meier curve and the survival rates at 5, 10 and 12 years after HTO were calculated to represent survival.

Radiographic parameters, knee scores, and patients' characteristics were described (mean ± standard deviation [minimum–maximum]; *n* [%]). The Wilcoxon singed‐rank test was used to compare preoperative knee scores to postoperative score values at the final follow‐up for the knees who did not receive a KA at the final follow‐up. Knees with and without conversion to KA were compared regarding patient demographics as well as preoperative and postoperative alignment parameters using the Mann–Whitney *U*‐test. Furthermore, univariate logistic regression analyses (odds ratio [OR]) were first performed to assess the association between each potential predictor and the risk of conversion to KA. Predictors included patient characteristics (gender, age and BMI), preoperative subjective knee scores (Lysholm and IKDC), grade of osteoarthritis in the medial and lateral compartments, and preoperative and postoperative alignment parameters (mTFA, mLDFA, MPTA and JLCA). These variables were then used to calculate a multivariate logistic regression model. A stepwise backward elimination approach was applied, sequentially removing the least significant variables. Adjusted OR were calculated and statistical significance was set at *p* < 0.05).

Statistical analysis was performed using JMP® (SAS Institute Inc., JMP®, Version 13.0.0) and STATA® (Stata Corporation, 15.0). The level of significance was set at 0.05 for all statistical tests.

## RESULTS

### Patient cohort and characteristics

From initial 120 knees from 112 patients, 95 knees could be followed‐up after 12 years postoperatively (Figure 1). Patient's demographics, preoperative and postoperative alignment (6 weeks postoperatively), as well as preoperative knee function are summarised in Table 1.

**Table 1:** Patients' demographics, preoperative alignment parameters, as well as subjective knee function in terms of mean ± SD (minimum–maximum); median (minimum–maximum); *n* (%).

	Total cohort (*n* = 95)
Age [years]	47.0 ± 7.6 (19.2–61.7)
Gender	
Female	28 (29.5%)
Male	67 (70.5%)
Weight [kg]	90.4 ± 17.1 (57–150)
Follow‐up period [years]	12.9 ± 0.8 (12.0–15.1)
Preoperative scores	
IKDC Score	48.6 ± 16.6 (14.9–93.1)
Lysholm Score	54.7 ± 20.9 (14–91)
Tegner Score	3 (1–10)
Preoperative alignment parameters	
mTFA	−5.1 ± 2.3 (−12 to −0.2)
mLDFA	89.1 ± 2.0 (84.4–96.9)
mMPTA	86.1 ± 2.7 (80.3–95.3)
JLCA	2.5 ± 1.7 (−1.2 to 7.0)
Grade of osteoarthritis	
Medial compartment	0 (0%), I (1.1%), II (65.1%), III (22.2%), IV (1.6%)
Lateral compartment	0 (7.9%), I (60.3%), II (31.7%), III (0%), IV (0%)
Patellofemoral compartment	0 (15.8%), I (63.5%), II (17.5%), III (3.2%), IV (0%)

Abbreviations: IKDC, International Knee Documentation Committee (Score); JLCA, joint line convergence angle; mFTA; mLDFA, mechanical lateral distal femur angle; mMPTA, mechanical medial proximal tibia angle; mTFA, mechanical tibiofemoral angle; SD, standard deviation.

### Conversion rate

**Figure 2:**
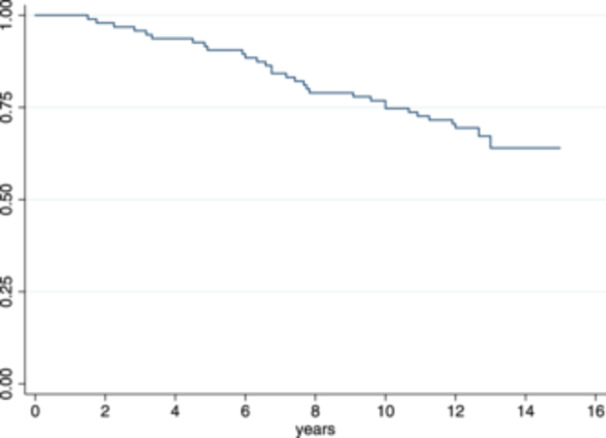
Kaplan–Meier Curve showing the survival of medial opening‐wedge high tibial osteotomy (HTO).

31 of 95 knees underwent conversion to knee arthroplasty (32.6%) on average after 7.3 ± 3.3 (1.5–13.0) years after the HTO surgery. Twenty‐nine knees were converted to a total knee arthroplasty, two knees to a unicompartmental knee arthroplasty (conversion after 10.0 and 4.9 years). The survival is shown in Figure 2 using the Kaplan–Meier curve. 64 HTOs were not converted to KA and were followed up on average 12.9 ± 0.8 (12.0–15.1) years postoperatively. The 5‐year, 10‐year, and 12‐year survival rates were 88.2%, 76.3% and 69.7%, respectively.

The comparison between knees without a conversion to KA and patients who received a KA during the follow‐up period revealed significant differences:

The percentage of females was significantly higher in knees receiving a KA (*p* = 0.0056). Knees who received a KA had lower preoperative subjective knee scores. Knees receiving a KA had a significant higher preoperative mMPTA and a higher preoperative JLCA. Age, body weight, preoperative mLDFA and mTFA, and postoperative alignment parameters did not differ significantly between the groups (Table 2). The univariate logistic regression showed that female gender (OR 3.68; *p* = 0.006), lower preoperative knee function (IKDC: OR 0.96; *p* = 0.008; Lysholm: OR 0.96; *p* < 0.001) and higher preoperative MPTA (OR 1.20; *p* = 0.038) are significantly associated with higher odds for conversion to KA. Postoperative alignment parameters (mTFA, mLFDA and mMPTA) were not significantly association. In the multivariate logistic regression analysis, only preoperative Lysholm score remained independently associated with conversion to KA (OR 0.96; *p* < 0.001). Other variables were not significantly associated after adjustment.

**Table 2:** Difference between patients with and without conversion to KA (mean ± SD [minimum–maximum]; *n* [%]).

Parameter	Conversion to KA (*n* = 31)	No conversion to KA (*n* = 64)	*p*‐Value
Age [years]	48.4 ± 6.5 (35.3–58.0)	46.4 ± 8.0 (19.2–61.7)	n.s.
Gender	Female: 53.6%	Female: 20.3%	0.0056*
Preoperative subjective knee function			
IKDC [%]	41.7 ± 14.6 (20.7–91.0)	51.8 ± 16.6 (14.9–93.1)	0.0016*
Lysholm [points]	43.0 ± 14.9 (22.0–78.0)	60.4 ± 21.1 (14.0–91.0)	<0.0001*
Preoperative alignment			
mTFA [°]	−5.0 ± 2.6° (−11 to −0.2)	−5.1 ± 2.2 (−12 to −0.3)	n.s.
mMPTA [°]	87.0 ± 2.9 (80.6–95.3)	85.7 ± 2.5 (80.3–91.2)	0.0305*
JLCA [°]	3.1 ± 1.7 (0.4–7)	2.2 ± 1.7 (−1.2 to 6.0)	0.0131*
mLDFA [°]	88.8 ± 2.3 (84.4–96.9)	89.2 ± 1.8 (85.2–93.8)	n.s.
Postoperative alignment			
mTFA [°]	1.7 ± 2.4 (−4.2–7.6)	2.0 ± 2.3 (−2.2 to 9.1)	n.s.
mMPTA [°]	92.9 ± 3.0 (86.1–98.1°)	92.7 ± 2.8 (87.6–101.7)	n.s.
JLCA [°]	2.6 ± 2.0 (0.3−6.5)	1.9 ± 1.7 (−0.7 to 9.1)	n.s.

Abbreviations: IKDC, International Knee Documentation Committee (Score); JLCA, joint line convergence angle; KA, knee arthroplasty; mFTA; mLDFA, mechanical lateral distal femur angle; mMPTA, mechanical medial proximal tibia angle; mTFA, mechanical tibiofemoral angle; SD, standard deviation.

* Statistical significance.

### Fulfilment of patient expectations and patient satisfaction

**Figure 3:**
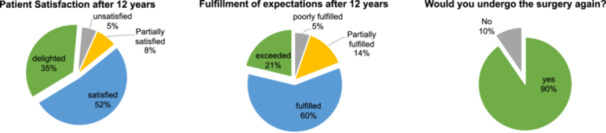
Fulfilment of patient expectations and patient satisfaction.

After 12 years, the majority were satisfied (52%) or even delighted (35%) about the surgical outcome (Figure 3). The expectations were fulfilled (60%) or exceeded (21%) (Figure 3), and 90% would undergo the surgery again. 96.8% of cases with no conversion to KA would undergo the surgery again. But even the majority who received a KA would undergo HTO surgery again (77.4%). Out of the 31 patients with conversion to KA, 67.7% (*n* = 21) answered that their expectations of HTO surgery were fulfilled, 9.7% (*n* = 3) exceeded and 6.5% (*n* = 2) partially fulfilled. Only 16.1% (*n* = 5) of the patients answered that their expectations were not fulfilled.

### Subjective knee function

**Figure 4:**
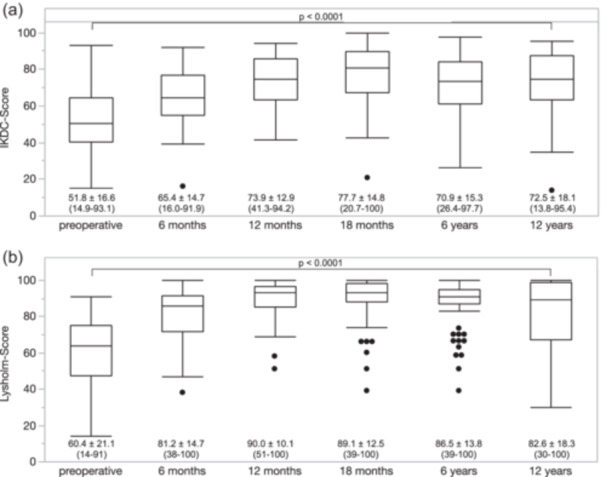
Subjective knee function measured with the IKDC Score (a) and the Lysholm score (b) over the 12‐year follow‐up period. Values are given as mean ± SD (min.–max.). IKDC, International Knee Documentation Committee (Score).

**Figure 5:**
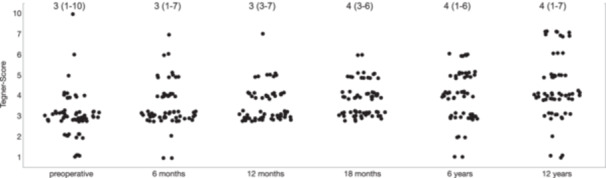
Tegner Activity level during the follow‐up period (median [minimum – maximum]).

The subjective knee function of knees who did not receive a KA improved after the surgery and remained higher at the final follow‐up (12 years postoperative) than preoperatively. The IKDC score was significantly (*p* < 0.0001) higher at the last follow‐up (72.5 ± 18.1 [13.8–95.4]) compared to preoperatively (51.8 ± 16.6 [14.9–93.1]) (Figure 4). The Lysholm score was 60.4 ± 21.1 (14.0–91.0) preoperatively and 82.6 ± 18.3 (30.0–100.0) postoperatively (*p* < 0.0001; Figure 4). The Lysholm score was, on average, the highest at 12 months postoperatively, and the IKDC Score was the highest at the 18 months follow‐up (Figure 4). Tegner activity scale showed that patients had a higher median activity level 12 years postoperatively (4 (1–7)) compared to the preoperative assessment (3 (1–10)) (Figure 5).

## DISCUSSION

The most important finding of the present study is that medial open wedge HTO is an effective surgery to treat patients with symptomatic medial compartmental OA of the varus knee in the long‐term. The conversion rate to knee arthroplasty was low with a 5‐year, 10‐year and 12‐year survival rate of 88.2%, 76.3 and 69.7%. The study demonstrated high levels of patient satisfaction, with long‐term expectations generally met. Furthermore, patients who did not undergo conversion to KA reported, on average, better subjective knee function at final follow‐up compared to their preoperative status.

In recent years, several studies reported long‐term survival rates following osteotomies around the knee. The survival rate of the present study is comparable to those reported in previous publications. For example, Darees et al. [6] reported a 10‐year survival rate of 88% in a cohort of 48 patients who underwent medial open‐wedge HTO using the TomoFix plate. Similarly, Bouguennec et al. reported a 5‐year survival rate of 93.0%, and a 10‐year survival rate of 74.0% [3]. A systematic review by Loke et al. [25] reported that the average rates of conversion to knee arthroplasty of 32 studies comprising 2840 patients were 4.5% at less than 5 years (95% CI, 2.3%–8.7% years), 8.3% at 5–10 years (95% CI, 5.7%–12.0% years) and 11.2% at more than 10 years (95% CI, 8.8%–14.2% years). Another systematic review by Ollivier et al. [32], which analysed 30 studies encompassing 7.087 HTO procedures, reported survival rates ranging from 86.0% to 100% at 5 years, 64.0% to 97.6% at 10 years, 44.0% to 93.2% at 15 years, and 46.0% to 85.1% at 20 years. However, in both systematic reviews most of the included studies were retrospective in design and report, in part, very high loss‐to‐follow‐up rates [5, 26, 31, 41]. In addition, there is considerable heterogeneity, particularly about the surgical techniques (opening vs. closing wedge HTO) and the implants used [5, 29, 31]. Consequently, the authors of these systematic reviews emphasised the need for prospective studies of higher quality and homogeneity in the future.

The present study comprises a homogeneous sample regarding surgical technique, implant, and follow‐up period, with a low rate of loss to follow‐up. Regarding modern indications, it should be noted that the present study reports long‐term outcomes based on surgeries performed between 2008 and 2011. In recent years, evidence has consistently shown that postoperative overcorrection of the mMPTA is associated with poorer functional outcomes. The univariate regression analysis of the present study could not show a significant association between postoperative MPTA and higher odds of conversion to KA. Furthermore, it has become increasingly recognised that corrective osteotomy should be performed at the site of deformity to avoid postoperative pathological joint angles [38]. In the present study, preoperative alignment measurements revealed varus deformity up to −19° varus. Furthermore, patients with mMPTA above 90° and mLDFA up to 97° were indicated for medial open wedge HTO. Nowadays, a deformity analysis with these alignment parameters would lead to surgical procedures such as a double level osteotomy or closed wedge distal femoral osteotomy [1, 10, 20, 34]. Furthermore, the cohort comprised patients with over‐ and undercorrection. The postoperative mTFA was in 7 cases still more than −1° varus, and in 19 cases ≥ 4° valgus. Recently, for the mTFA, an individual and more neutral correction is recommended [9, 35]. Ultimately, some patients had postoperative pathological mechanical joint line angles. Nowadays, more physiological deformity angles are targeted to minimise shear stress in the medial compartment as well as postoperative unintended alignment changes [4, 24, 30]. Despite these modifications to enhance the postoperative outcome following HTO surgery, the present study demonstrated high survival rates following HTO. Post‐hoc analysis of the present patient cohort with radiographic changes over time is planned.

The use of survival rate as a measure of success for HTO has been a topic of debate, as the absence of conversion to KA does not necessarily equate to a satisfactory or favourable patient outcome [8, 31]. Especially in younger patients, the decision to convert to arthroplasty is often placed with restraint by both the patient and the surgeon, even in the presence of pain or limited function. The present study showed that HTO can result in high patient satisfaction—not only among those who did not require conversion to KA, but even among those who eventually did. The majority of patients would choose to undergo the surgery again. The high level of patient satisfaction observed in this study aligns with previously published findings, as summarised in the systematic review by Ollivier et al. [30]. Across the included studies, between 77% and 98% of patients reported satisfaction after a mean follow‐up of more than 10 years. In a retrospective study by Hui et al., 93% of patients without conversion to knee arthroplasty and 68% of those who underwent revision were satisfied with the outcome. Overall, 84% of the included 394 patients who underwent lateral closing wedge HTO indicated they would choose to undergo the procedure again [17]. This consistently high level of satisfaction is particularly noteworthy given the high expectations patients typically have of osteotomies around the knee regarding return to work, pain relief, and functional restoration [13].

In their review, Webb et al. evaluated postoperative changes in functional outcomes after HTO. The included studies showed improvements in most patients regarding pain reduction, symptom relief, knee function, and return to activity [42]. The present study confirms these findings. Notably, patients without conversion to KA exhibited higher functional knee scores 12 years postoperatively compared to their preoperative baseline. This highlights the long‐term effectiveness of HTO surgery in patients with medial compartment knee OA.

Several factors have been shown to improve outcomes following medial open‐wedge HTO, including thorough preoperative planning, standardised surgical techniques, and the use of angular stable implants. Various studies have investigated predictive factors: Batailler et al. [2] identified age under 55, BMI under 25 kg/m², incomplete joint space narrowing, and female gender as favourable prognostic factors. Miettinen et al. [27] conducted a risk factor analysis using a Cox regression model, which did not identify age, gender, body weight, type of osteotomy, bone grafting or substitution, preoperative mTFA, or OA grade as significant risk factors. In contrast, Keenan et al. [22] showed female gender and older age as independent risk factors for conversion to KA. The analysis of the present study showed that female gender, lower preoperative subjective knee function and higher preoperative MPTA are risk factors for conversion to KA. However, in the multivariate regression model only lower preoperative subjective knee function was independently associated with conversion to KA. The comparison of patient characteristics between patients with and without conversion to KA showed that lower preoperative knee function and the percentage of females were significantly higher in patients receiving a KA. Furthermore, patients receiving a KA had in average a higher preoperative MPTA and a higher JLCA. HTO surgery was performed on different degrees of medial compartment OA and differing severities of limb deformity. However, there were no significant differences between the two patient groups in terms of preoperative mLDFA and mTFA, postoperative mTFA and MPTA, or the extent of preoperative cartilage damage in the medial compartment.

The present study has several limitations. The cohort comprised a heterogeneous patient sample with different ages, differently pronounced and localised deformities, and grades of OA. This might bias the conversion rate to KA. In contrast, the surgical technique and the implant used were applied consistently across the entire cohort, which strengthens the validity of the results.

Furthermore, the patient's demands and activity level are heterogenous as outlined by a large range in the preoperative and postoperative Tegner activity scale. Most patients took part preoperative and postoperatively on recreational sporting activities. Compared to the general population of the same age, the patient cohort is slightly above‐average BMI, but a generally reduced demand for knee function and knee symptoms cannot be assumed [16].

Of the initially included 120 knees, the present study achieved a high follow‐up rate of 79.2% despite the long‐term follow‐up period. However, due to the study design, no conclusions can be drawn regarding the optimal target for postoperative alignment, nor can the outcomes be compared with alternative surgical treatments such as unicompartmental knee arthroplasty in patients with medial compartmental OA. Another limitation is that the knee function immediately prior to the indication for KA was not assessed. Furthermore, the specific indications and reasons for conversion to KA remain unclear.

## CONCLUSION

Medial open wedge HTO is an effective surgical treatment for patients with medial compartmental OA and varus knee alignment. The conversion rate to KA remained low at 12 years postoperatively. Overall, the procedure demonstrated high patient satisfaction and long‐term fulfilment of patient expectations.

## AUTHOR CONTRIBUTIONS

Ahrend, Petzold and Herbst conducted the 12‐year follow‐up and examined the patients. Ahrend & Herbst wrote the mansucript. Schröter initiated the study. Schröter, Ihle, Ahrend, Herbst were responsible for supervision of the follow‐ups. All authors revised the initial mansucript.

## CONFLICT OF INTEREST STATEMENT

The authors declare no conflicts of interest.

## ETHICS STATEMENT

Please include the name of the institutional review board (IRB) and the approval number: The study was approved by the local ethics committee of the University of Tübingen (10/2008BO2, 409/2017BO2, and 488/2014BO2).
